# Is *Chlamydia* to Blame for Koala Reproductive Cysts?

**DOI:** 10.3390/pathogens10091140

**Published:** 2021-09-04

**Authors:** Samuel Phillips, Peter Timms, Martina Jelocnik

**Affiliations:** Genecology Research Centre, University of the Sunshine Coast, Sippy Downs, QLD 4556, Australia; ptimms@usc.edu.au (P.T.); mjelocni@usc.edu.au (M.J.)

**Keywords:** koala, *Chlamydia*, reproductive cysts

## Abstract

A significant threat to koala populations is infection from *Chlamydia*, which results in disease and death. Wild koalas with *Chlamydia* infections are admitted to wildlife hospitals and treated with antibiotics; however, up to 50% of koalas that present to wildlife hospitals do not survive. A major contributor to high mortality is the development of reproductive cysts, resulting in female infertility and euthanasia. However, the diagnosis of reproductive disease is limited to ultrasound with no further investigations. This communication highlights reports of histological and microbiological findings, the accuracy of ultrasound to necropsy reports and other possible causes for reproductive cyst development previously reported in other hosts. Our conclusions identify a significant knowledge gap in the aetiology of koala reproductive cysts and highlight the urgent need for future investigations.

The koala (*Phascolarctos cinereus*) is one of Australia’s most recognised native animals. Over the last 200 years, koala population levels have plummeted by approximately 90%, with some southern populations becoming locally extinct [1]. Unfortunately, koala population levels have continued to decline due to multiple natural and anthropogenic factors, and in 2012 the koala was listed as a threatened species under the Australian Federal Environment Protection and Biodiversity Conservation Act 1999. The largest threat to koalas is habitat destruction, which has resulted in up to 80% of the koala natural habitat (eucalyptus forest) being cleared for human population growth and farming [2]. In addition to habitat destruction, a recent report of 3590 koala deaths within a single wildlife hospital over a 10-year period identified the top three reasons for koala fatalities as traffic accidents (36.4% of deaths), overt signs of disease (33.6% of deaths) and dog attacks (13.8% of deaths) [3]. 

A major threat to the health and wellbeing of koalas is the bacterium *Chlamydia pecorum*. *C. pecorum* is the leading cause of disease in koalas, causing high morbidity and mortality [4]. *C. pecorum* infections in free-ranging koalas in southeast Queensland are endemic, with clinical (chlamydial) disease observed in 30% of surveyed animals [5,6]. Many sub-populations of koalas are on the brink of local extinction due to chlamydial disease, with estimated declines in some populations of between 55% and 80% between 1996 and 2014 [7]. A failed scheme developed in Victoria in an attempt to increase koala populations reported that translocations of healthy, *Chlamydia*-free koalas into areas of high *Chlamydia* burden resulted in increased chlamydial disease in the translocated koalas, leading to continued decline of the population [8]. 

*C. pecorum* causes urogenital and conjunctival infections in koalas with the potential to result in suffering and long-term debilitating disease [9,10,11]. Infections associated with the eyes cause conjunctivitis, which in severe cases results in scarring and blindness (keratoconjunctivitis) [9,10,11]. Infections associated with the urogenital tract can cause inflammation resulting in “wet bottom”, and the development of cystitis, which in extreme cases results in ureter ascension and nephritis [9,10,11].

Infections and disease of the urogenital tract account for approximately 87% of all infections in some koala populations [6,9,12]. *Chlamydia* infections in the koala are treated with antibiotics for extended periods of time (up to 45 days) resulting in complete bacterial clearance [9,13]. Unfortunately, some wild koalas develop chronic, advanced disease before being found and brought to wildlife hospitals, furthermore, antibiotic treatment failure occurs, resulting in disease progression and eventual death [14]. Reproductive tract disease can progress in female koalas and result in reproductive cyst development and presumed infertility [9]. Reproductive cysts have been reported to develop during and after antibiotic treatment, even after microbiological cure. There are also reports of reproductive cysts decreasing to below detectable levels in the absence of treatment. Currently, it is not known why or how reproductive cysts in koalas develop, or why some resolve. 

Reproductive cysts in koalas are commonly referred to as paraovarian or bursal cysts and are presumed to result in infertility of the koala. However, according to Jubb, Kennedy and Palmer’s Pathology of Domestic Animals, ovarian cysts in other animal species are categorised into several different types. Cystic rete tubules, paraovarian cysts, inclusion cysts and anovulatory cysts of follicular or luteinizing type are difficult to determine under ultrasound and are never associated with *Chlamydia* infection [15]. Cystic rete ovarii have been identified in 76% of female guinea pigs and are most commonly (66–77%) non-functional [16]. Inclusion cysts are usually diagnosed in older animals, are related to increased ovulation, and often disappear over time [17]. Anovulatory cysts are due to secondary follicles that fail to ovulate and are usually reabsorbed without complication [18]. Paraovarian cysts are cysts that develop close to the ovaries and the fallopian tubes but never attach. They are usually associated with the surrounding ligaments and are also known as a paratubal cysts or hydatid cysts [19]. 

Host immunological responses to *Chlamydia* infections have been extensively studied in the mouse model where it is established that both cell-mediated immune responses involving the recruitment of macrophages, dendritic cells, natural killer cells and CD4/CD8 T cells and a humoral immune response involving both plasma IgG and mucosal IgG and IgA are required for complete bacterial clearance [20,21]. It has also been established that IFNγ restricts the growth cycle of *C. trachomatis* by depleting tryptophan through the indoleamine 2,3-dioxygenase (IDO) pathway [22,23,24]. However, the known specific immunological process by which the koala immune system responds to a *C. pecorum* infection contains significant gaps [25]. Recently, in vitro studies of koala PBMCs stimulated with *C. pecorum* elementary bodies (EBs), identified the upregulation of specific cytokines, previously shown in mouse trials to be involved in *Chlamydia* clearance. Cytokines TNF alpha, IL10, IFNγ and IL17 were all shown to be highly upregulated after *C. pecorum* EB stimulation [26,27,28]. Interestingly, in vitro studies have recently identified that *C. pecorum* (livestock strains) are resistant to IFNγ responses alone, a trait attributed to the near intact tryptophan biosynthesis genes [29]. 

It is unknown if reproductive cysts in koalas are directly related to chlamydial infections of the upper reproductive tract or due to inflammation and immunological responses to chlamydial infections of the lower reproductive tract or something entirely separate to *Chlamydia* infection. 

The development of reproductive disease in koalas has been sporadically reported since 1981 with surprisingly concordant findings. Overall, bursal cysts form in the fallopian tubes of koalas and can involve one or both tubes. Sizes can vary between 1 cm and 15 cm, and the cysts are fluid-filled. The appearance of this fluid has been reported to differ between koalas and to be straw coloured, red-brown or turbid, with differences in viscosity also reported [30,31]. Histological findings report that the ovaries are usually unaffected with a normal bursal lining, although some studies have reported extensive fibrous adhesions in the ovary and bursal wall space of female koalas, indicating that ovarian disease is possible [31,32,33]. Histological reports of fallopian tube inflammation indicate accumulations of plasma cells, lymphocytes and macrophages in the submucosa and polymorphs and macrophages in the lumen [30,31]. Other studies have reported similar findings of infiltrative immune cells from different upper and lower urogenital tissues [34,35]. A study in 1984, using radiographic techniques, reported that 43% of koalas (101/237) across four states of Australia were found to have reproductive cysts with unknown aetiology (although *Chlamydia* was isolated) [36]. These initial reports all failed to conclusively indicate an aetiology for reproductive cyst development, although they speculated that there was the involvement of an infectious agent. The first attempts to identify an aetiology used immunohistochemical stains for *Chlamydia* from upper reproductive tract tissue of female koalas and identified the presence of *Chlamydia* in 10 koalas with active reproductive inflammation [34]. However, the same study also reported an absence of *Chlamydia* in koalas with tubal occlusions and high anti-chlamydial antibody titers, leading the authors to question the causal connection between ovarian cysts in the koala and chlamydial infection of the reproductive tract [34]. A further study of koalas from the South Australian Mount Lofty ranges performed histological examination on pariovarian cysts from a single koala described the cysts as thin-walled and fibrocollagenous with infrequent papilliform projections of dense fibrovascular connective tissue [37]. The same study did not report on the presence of chlamydia within the cysts nor perform any immunological tests [37]. 

The first reports of reproductive cyst development in koalas without inflammation of the lower genital tract were in 1989 when reproductive cysts were identified in five out of nine koalas that were suffering from cystitis but did not display inflammation within the lower reproductive tract [38]. Other than this report, there have been no other published studies on spontaneous development of reproductive cysts in koalas. Furthermore, there are also no reports of *Chlamydia*-specific studies from upper reproductive tract tissues or cystic fluid, and no reports of possible treatment regimens to clear disease. Considering that the Queensland government guidelines on koala treatment indicate euthanasia in cases of suspected infertility (proposed to limit habitat competition with fertile koalas) [39], the development of improved treatment regimens to combat this disease would significantly improve koala health outcomes and the chance of survival. 

Several strategies are used by veterinarians to diagnose *Chlamydia* disease in the koala. The urogenital disease state of a koala caused by *C. pecorum* infection is assessed through ultrasound scanning of the internal urogenital organs (i.e., bladder, prostate, kidneys, ovaries and ureter tubes), clinical inspection of the koala’s rump for evidence of a ‘wet bottom’ and cytological examination of urine sediment [9]. The overall health of a koala is measured using several markers including hydration status (measured by observing the tactility of the skin), gut fill (observed through abdominal palpation), lymph node enlargement (determined by palpation) and body condition scoring (measured through muscle measurement of the scapula muscles) [9]. Unfortunately, the identification of reproductive disease is limited to ultrasound imaging. This approach lacks the ability to determine the differences between chronic and acute disease. Furthermore, a recent analysis of 38 ultrasound reports and necropsy findings identified that in 44% of cases necropsy failed to conform with ultrasound diagnosis [40]. 

Of course, reproductive cyst development is not limited to female koalas, with naturally occurring reproductive cysts identified in rats [41,42], cynomolgus and rhesus monkeys [43,44], cows [45,46,47], dogs [48], pigs [49] and water buffalo [50]. Interestingly, none of these presentations were attributed to an infectious agent, but were instead related to hormone imbalance induced by many different environmental and hormonal factors, including repeated cold stress [41], increased light stress [42], increased reactive oxygen species [50,51,52,53], decreased melatonin [42] and ingestion of a high soy-based diet [54]. Furthermore, Rubio and colleges reported that the ovarian bursa of dogs can harbour bacterial colonisation in the absence of disease [55]. 

Women between the ages of 15 and 44 have a 5–10% risk of developing polycystic ovary syndrome (PCOS), a disease related to hormonal disturbances, such as hyperandrogenism [56]. It appears that the development of chlamydial induced reproductive cysts outside the koala have only been sparingly reported. A single study in cows identified ovarian cysts in the presence of *Chlamydia* infections, although the authors state that “it is not likely that chlamydia is of causal relevance for ovarian cysts” [57]. Another study in 1994 investigating *Chlamydia* infections in women also identified a significant correlation between the presence of anti-chlamydia IgG and tubular infertility, with 2 out of 10 tubal biopsies confirming *Chlamydia* infection [58]. The development of upper reproductive pathology, not involving the ovaries or fallopian tubes, has been described in mice and guinea pigs. Mice have been shown to develop hydrosalpinx, uterine cysts and dilated glandular ducts infected with *Chlamydia* (direct inoculation or urogenital infection) [59]. Guinea pig studies also identified a correlation between estradiol supplements and increased chlamydial disease, specifically, ascending infections resulting in endometritis, cystic salpingitis and cystitis [60]. 

There are clear reports indicating a difference in the physical appearance of cysts developing in koalas, and evidence of other non-infectious aetiological agents in other hosts. Considering this, future research efforts should be focused on identifying all aetiological agents for the development of koala reproductive cysts with an aim of developing new treatment regimens to allow for the recovery of female koalas. This would significantly improve the population growth of koalas with more koalas of reproductive age being released back into the wild. 
